# The Role of Lymph Node Dissection for Non-Metastatic Renal Cell Carcinoma: An Updated Systematic Review and Meta-Analysis

**DOI:** 10.3389/fonc.2021.790381

**Published:** 2022-01-12

**Authors:** Xu Shi, Dechao Feng, Dengxiong Li, Facai Zhang, Wuran Wei

**Affiliations:** Department of Urology, Institute of Urology, West China Hospital, Sichuan University, Chengdu, China

**Keywords:** lymph node dissection, non-metastatic renal cell carcinoma, high-risk renal cell carcinoma, overall survival, meta-analysis

## Abstract

**Introduction:**

To compare the survival benefit of nephrectomy with or without lymph node dissection (LND) for non-metastatic, especially for high-risk renal cell carcinoma (RCC) patients by investigating different survival evaluation indicators.

**Evidence Acquisition:**

Eligible studies were identified until September 2021, through common databases including PubMed, the Cochrane Library, Embase and China National Knowledge Infrastructure (CNKI) on RCC and LND without language restriction. Data analysis was performed through Stata software, version 16.0 (Stata Corp., College Station, TX, USA).

**Evidence Synthesis:**

22 articles were included in this meta-analysis. For non-metastatic RCC, performing LND comitantly with nephrectomy did not change the overall survival (OS) of patients of all T stages [hazard ratio (HR)=1.10, 95%CI: 0.95-1.27] and also for T2+NxM0 patients (HR=0.88, 95%CI: 0.68-1.14) as well as for T3+NxM0 patients (HR=0.95, 95%CI: 0.61-1.50). At the same time, cumulative meta-analysis has shown that the survival benefit of LND has a significant declining trend since 1979. However, it is worth noting that the operation of LND presented as a risk factor for cancer specific survival (CSS) (HR=1.22, 95%CI: 1.05-1.43).

**Conclusions:**

Latest evidence indicated that LND might not be suitable for all non-metastatic RCC patients, especially in the current situation of various non-invasive examinations for judging lymph node metastasis and adjuvant treatments. On the contrary, excess LND could damage the survival of patients.

**Systematic Review Registration:**

This study is registered as PROSPERO CRD42021271124.

## Introduction

The incidence of renal cell carcinoma (RCC) is increasing in the past 20 years, which may due to the widespread promotion of abdominal imaging technology (1, 2). Whether or not to perform lymph node dissection (LND) during nephrectomy is still unclear. At present, it is mostly believed that LND should be considered only when lymph node metastasis and/or swollen lymph nodes are found in preoperative imaging examination or during surgery, and its significance is mainly restricted to provide accurate clinical staging rather than survival benefit (3, 4). But what is of vital importance for clinicians is to assess whether RCC of each stages require LND. Recent research controversy is mainly focused on whether the clinical benefits of LND in medium-to-high-risk patients can be obtained. In this regard, we conduct a systematic review and meta-analysis, covering a wide range of articles focusing on the impact of LND on the survival of non-metastatic renal cell carcinoma patients since 1979.

### Evidence Acquisition

#### Eligible Criteria for Study Selection

##### Types of Studies

Only randomized controlled studies (RCT), prospective and retrospective studies are all included in this article. Animal studies, case reports, editorial comments and reviews are excluded. The patients, intervention, comparison, outcome, and study design (PICO) approach were used to define study eligibility.

##### Types of Patients

Patients included those diagnosed with non-metastatic RCC regardless of pathological type, tumor stages or grades. Diagnoses must be based on pathological findings.

##### Types of Interventions

Interventions included nephrectomy with LND, as well as other surgical treatment including NSS or palliative surgery, without restrictions on whether the patient has received chemotherapy and other adjuvant treatments, and the template of LND was specified by each unit.

##### Types of Comparisons

Comparisons included nephrectomy or other surgical treatments without LND. The primary outcome was overall survival (OS). Secondary outcomes were other survival indicators including progression-free survival (PFS) and cancer specific survival (CSS).

##### Types of Outcome Measures

The primary outcome was overall survival (OS). Secondary outcomes were other survival indicators including progression-free survival (PFS) and cancer specific survival (CSS).

### Literature Research Strategy

The searched databases included: PubMed, the Cochrane Library, Embase and China National Knowledge Infrastructure (CNKI). The last update date was September 2021, without language restriction. Our search strategy was as follows: ((((((lymphadenectomy) OR (lymph-node dissection)) OR (lymph node dissection)) OR (LND)) OR (node dissection)) AND ((((Neoplasm*[Title/Abstract]) OR (Cancer*[Title/Abstract])) OR (carcinoma [Title/Abstract])) OR (tumor [Title/Abstract]))) AND ((Kidney [Title/Abstract]) OR (Renal [Title/Abstract])). Published articles and ePub ahead of print within PubMed, the Cochrane Library, Embase and China National Knowledge Infrastructure (CNKI) were included. Non-peer-reviewed publications and unpublished studies and abstracts were excluded. There were no restrictions on the baseline characteristics including the pathological type of RCC and the template for LND. At the same time, the “related articles” function and the reference lists of the retrieved articles was also screened to try to include all the documents in this field. Two authors (XS and DCF) respectively conducted a search according to the Preferred Reporting Items for Systematic Reviews and Meta-Analyses (PRISMA) and merged the information (5). Any objections were decided by another author (WRW) after discussion.

## Data Extraction

The two authors (XS and DCF) separately extracted the following content: author, publication year, language, country, institute, intervention and control measures, tumor grade and stage, as well as survival indicators. Dispute was decided by another author (WRW) after discussion. If there was no mention of exact figure of hazard ratio (HR) in the full-text and was presented in the form of a Kaplan-Meier survival curve, we used Engauge Digitizer software to extract the HR value and its 95% confidence interval (CI) (6). Engauge Digitizer is a tool for more accurately extracting digital information from published data presented in graphical form (7). It should also be emphasized that in the “*Results*” section of this article, patients with high-risk RCC were defined as T3+M0. But a precise definition of high-risk RCC patient group that is truly suitable for LND was mentioned in the “*Discussion*” section, which remained an interesting and important topic.

## Study Quality Assessment

Two authors (XS and DCF) independently used the Newcastle Ottawa Scale (NOS) to score the risk of bias if the included observational studies (8). Articles with a NOS score of seven or more of nine stars are considered high quality. We also evaluated the bias for the only RCT using the Cochrane Collaboration’s Risk of Bias (RoB) tool in Review Manager software (https://community.cochrane.org/help/tools-and-software/revman-5) from 7 domains: random sequence generation (selection bias); allocation concealment (selection bias); blinding of participants and personnel (performance bias); blinding of outcome assessment (detection bias); incomplete outcome data (attrition bias); selective reporting (reporting bias); other bias (such as funding sources), the results of which was shown in **Figure 3C**. In addition, the two authors (XS and DCF) independently graded all the included literature using the Oxford Centre for Evidence-Based Medicine criteria (9).

## Data Analysis

tata software, version 16.0 (Stata Corp., College Station, TX, USA) was used for data analysis. We combined the HR of articles with the same type of patients and the same survival indicator, and calculated the 95% CI. We assessed heterogeneity using Q test and I² test. The random effects model was used to analyze the data and sensitivity analysis was performed to detect the source of heterogeneity. We conducted a subgroup analysis of the survival outcomes of LND in T2+M0 and T3+M0 patients. We also showed the results of the sensitivity analysis to give a relatively robust outcome. A funnel plot and Egger’s test was used to screen for potential publication bias. After that, we also conducted cumulative meta-analysis according to the year. We also tested the heterogeneity of the meta-analysis in a 10-year span and we pooled the results using two-sided P-value of <0.05 for each outcome. When the data of two articles were duplicated or came from an overlapping period of time, we choosed the one with the largest sample size and the latest publication. The article of Farber et al. included a database from NCDB (2010-2014) with a sample size of 19,500 patients (10). While the database of Bacic et al. was from NCDB (2004-2013) with a sample size of 69,477 patients (11). Therefore, the study of Farber et al. was excluded from our analysis.

## Results

### Literature Search Results

**Figure 1:**
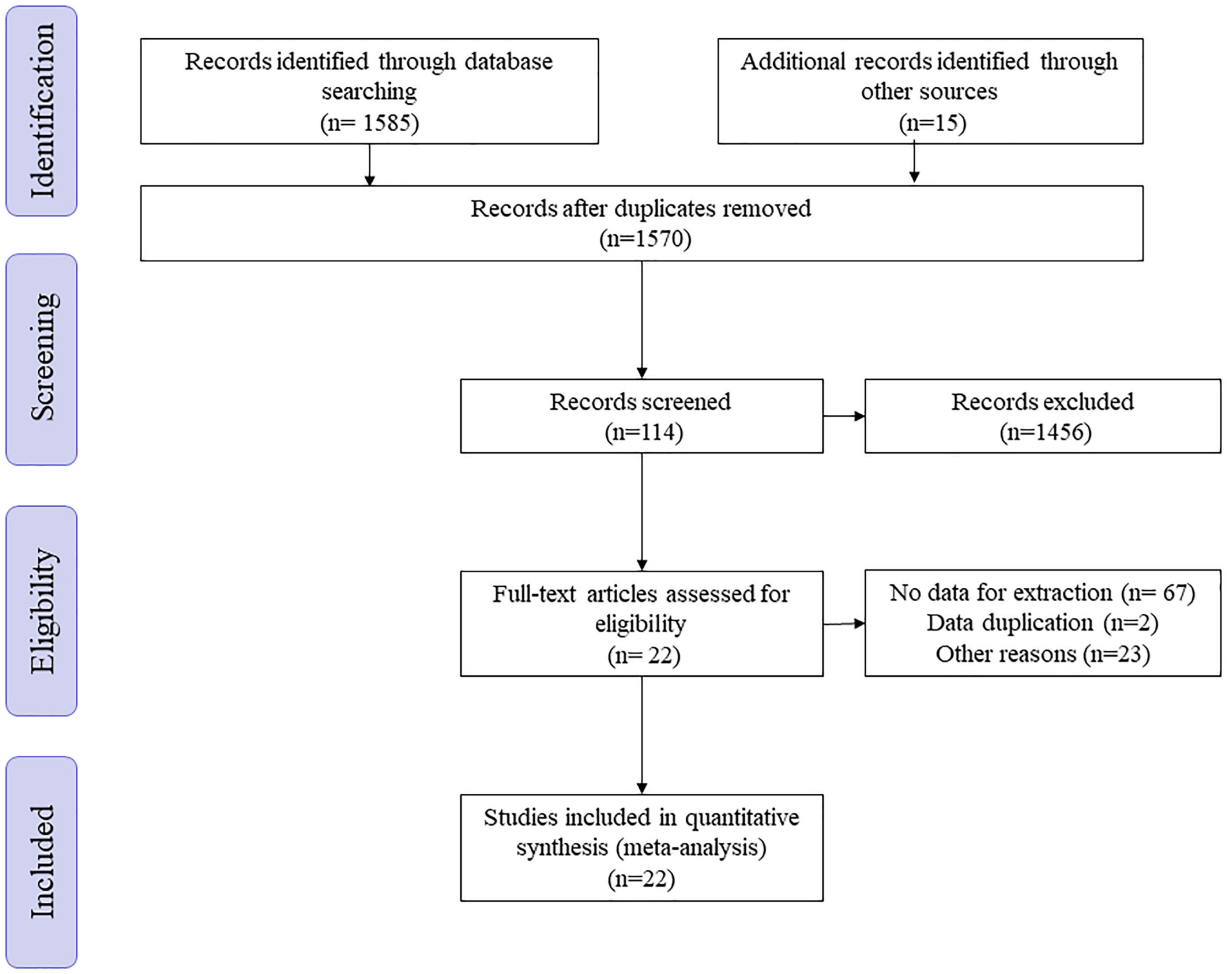
The study selection process.

A total of 1585 articles was found through keyword search through systematic research of PubMed, the Cochrane Library, Embase and China National Knowledge Infrastructure (CNKI) up to September 2021 without language limitation. After removing duplicates, 1570 articles were left. By reading the title and abstract, 1456 articles were removed. Of the remaining 114 articles, after reading the full text, 92 articles were excluded because of missing data or duplication of the database (**Figure 1**).

The baseline characteristics of the included 22 articles with a total of 135514 patients (29044 with LND and 106471 without LND) from 7 countries were presented as the following table, including one RCT as well as one prospective study, the rest of which were retrospective studies (**Table 1**). The same article by Li et al. contained two unique databases from China and the United States, so Li (2019, 1) and Li (2019, 2) were used to distinguish them in the table (21). The only RCT article (EORTC 30881) so far was from Blom et al. (29). 17 researches included the impact of LND on the OS of patients with non-metastatic RCC (11–24, 26–28). Three databases from two articles contained survival information of T3+M0 patients (21, 25). There was also one article covering the survival information of the T2+M0 patients’ population as well (27). In addition, the outcome indicators of 3 articles were PFS (21, 23, 29) and 4 articles were CSS (21, 22, 30, 32).

**Table 1:** The basic characteristics of the included studies.

Author (year)	Language	Country	Institute (Duration)	Study design (Level of evidence)	NOS score	Staging/Grading tool	Intervention	Case	Pathological stage	Tumor grade
Golimbu (12)	English	USA	New York University (1970-1982)	Retrospective study (2b)	6 of 9	Robson staging	LND	52	I:21; II:6; III:25	NM
							Non-LND	141	I:62; II:42; III:31	NM
Kobayashi (13)	Japanese	Japan	Gunma University (1961-1989)	Retrospective study (2b)	6 of 9	Robson staging	LND	39	I/II	NM
							Non-LND	63	I/II	NM
Katagiri (14)	Japanese	Japan	Niigata University and related hospitals (1987-1996)	Retrospective study (2b)	6 of 9	NM	LND	173	NM	NM
							Non-LND	44	NM	NM
Wang (15)	Chinese	China	General Hospital, Benxi Steel and Iron Company (1980-1995)	Retrospective study (2b)	6 of 9	TNM(UICC)	LND	82	NM	NM
							Non-LND	149	NM	NM
Minervini (16)	English	Italy	University of Pisa (1990-1997)	Retrospective study (2b)	7 of 9	TNM(UICC)1997	LND	49	T1:31; T2:11; T3:5; T4:2	G1:13; G2:34; G3:4
							Non-LND	108	T1:75; T2:201; T3:81; T4:5	G1:36; G2:62; G3:10
Pantuck (17)	English	USA	University of California School of Medicine	Retrospective study (2b)	8 of 9	NM	LND	433	NM	NM
							Non-LND	365	NM	NM
Sullivan (18)	English	Canada	Vancouver General Hospital	Retrospective study (2b)	6 of 9	Robson staging	LND	15	II:15	NM
							Non-LND	9	II:9	NM
Russo (19)	English	USA	Memorial Sloan-Kettering Cancer Center (1989-2004)	Retrospective study (2b)	8 of 9	AJCC 2017	LND	487	T1a:114; T1b:155; T2:218	NMN
							Non-LND	1116	T1a:677; T1b:294; T2:145	M
Ristau (20)	English	USA	ASSURE (ECOG-ACRIN 2805) (2006-2010)	Prospective study (2a)	8 of 9	Fuhrman\AJCC	LND	701	T1:16; T2:187; T3:330; T4:161	G1:23; G2:149; G3:519; G4:10
							Non-LND	1241	T1:32; T2:418; T3:566; T4:215	G1:159; G2:331; G3:737; G4:14
Li (21, 1)	English	China	Nankai University (2006-2013)	Retrospective study (2b)	8 of 9	2018-TNM/AJCC、WHO/ISUP	LND	67	M0: 30; M1:37	WHO/ISUP 1-2: 17; WHO/ISUP 3-4: 28
							Non-LND	67	M0: 26; M1:41	WHO/ISUP 1-2: 23; WHO/ISUP 3-4: 28
Li (21, 2)	English	USA	TCGA (2010-2014)	Retrospective study (2b)	8 of 9	2018-TNM/AJCC、WHO/ISUP	LND	NM	NM	NM
							Non-LND	NM	NM	NM
Bacic (11)	English	USA	the National Cancer Database (2004-2013)	Retrospective study (2b)	8 of 9	NM	LND	10078	pT1:2720; pT2:2348; pT3:4493; pT4:234; missing:283	G1:458; G2:3220; G3:3436; G4:1378; missing:1586
							Non-LND	59399	pT1:34990; pT2:8891; pT3:10726; pT4:226; missing:4566	G1:6172; G2:27777; G3:14225; G4:2471; missing:8754
Kokorovic (22)	English	Canada	The Canadian Kidney Cancer (2011-2019)	Retrospective study (2b)	8 of 9	NM	LND	812	pT1a:46; pT1b:113; pT2:110; pT3:513; pT4: 30	G1/2:259; G3/4:537
							Non-LND	1887	pT1a:380; pT1b:455; pT2:251; pT3:786; pT4: 15	G1/2:871; G3/4:971
Alekseev (23)	English	Russia	P.A. Herzen Moscow Cancer Research Institute	Retrospective study (2b)	6 of 9	NM	LND	369	cT1a:36; cT1b:118; cT2:90; cT3a:99; cT3b:24; cT4:2	NM
							Non-LND	174	cT1a:39; cT1b:74; cT2:34; cT3a:22; cT3b:5; cT4:0	NM
Schafhauser (24)	English	Germany	University of Erlangen-Nürnberg (1974-1993)	Retrospective study (2b)	7 of 9	NM	LND	531	pT1/2:223; pT3:297; pT4:11	G1/2:366; G3:159; GX:6
							Non-LND	305	pT1/2:156; pT3:149; pT4:0	G1/2:238; G3:64; GX:3
Siminovitch (25)	English	USA	Cleveland Clinic Fundation (1968-1978)	Retrospective study (2b)	7 of 9	NM	LND	41	T3a:17; T3b:24	
							Non-LND	7	T3a:2; T3b:5	
Yamashita (26)	English	Japan	NM	Retrospective study (2b)	6 of 9	NM	LND	13	NM	NM
							Non-LND	2	NM	NM
Feuerstein (27)	English	USA	Memorial Sloan-Kettering Cancer Center (1990-2012)	Retrospective study (2b)	8 of 9	AJCC	LND	334	T2:262; T3:49; T4:12	NM
							Non-LND	190	T2:84; T3:101; T4:5	NM
Zhi (28)	Chinese	China	The first Affiliate Hospital of Dalian Medical University (2013-2015)	Retrospective study (2b)	7 of 9	NM	LND	140	NM	NM
							Non-LND	112	NM	NM
Blom (29)	English	Europe	EORTC Data Center	Randomized Controlled Trial (1b)	**#**	1978 TNM	LND	383	T1:34; T2:221; T3:112	G0:11; G1:78; G2:156; G3:67; G4:2; GX:34
							Non-LND	389	T1:23; T2:242; T3:10	G0:11; G1:98; G2:152; G3:49; G4:2; GX:37
Gershman (30)	English	USA	Mayo Clinic and San Raffaele Scientific Institute (1990-2010)	Retrospective study (2b)	8 of 9	2010 AJCC 、WHO/ISUP	LND	1039	pT1a:299; pT1b:401; pT2a:182; pT2b:96; pT3a:321; pT3b:64; pT3c:23; pT4:12	G1:104; G2:668; G3:531; G4:95
							Non-LND	1398	pT1a:195; pT1b:301; pT2a:136; pT2b:71; pT3a:253; pT3b:54; pT3c:18; pT4:11	G1:70; G2:471; G3:413; G4:85
Gershman (31)	English	USA	Mayo Clinic (1990-2010)	Retrospective study (2b)	8 of 9	2010 AJCC、WHO/ISUP	LND	606	pT1a:36; pT1b:84; pT2a:81; pT2b:66; pT3a:218; pT3b:91; pT3c:13; pT4:16	G1:12; G2:154; G3:310; G4:130
							Non-LND	1191	pT1a:340; pT1b:352; pT2a:152; pT2b:65; pT3a:210; pT3b:41; pT3c:11; pT4:9	G1:75; G2:590; G3:460; G4:66
Marchioni (32)	English	USA	the Surveillance, Epidemiology, and End Results database (2001-2013)	Retrospective study (2b)	8 of 9	Fuhrman	LND	41644	NM	NM
							Non-LND	38114	NM	NM

LND, lymph node dissection; NOS, Newcastle-Ottawa Scale; NM, not mentioned.

#: The quality evaluation of RCT article by Blom et al. can be found in **Figure 3C**.

### Survival Benefit of LND for Non-Metastatic RCC Patients

**Figure 2:**
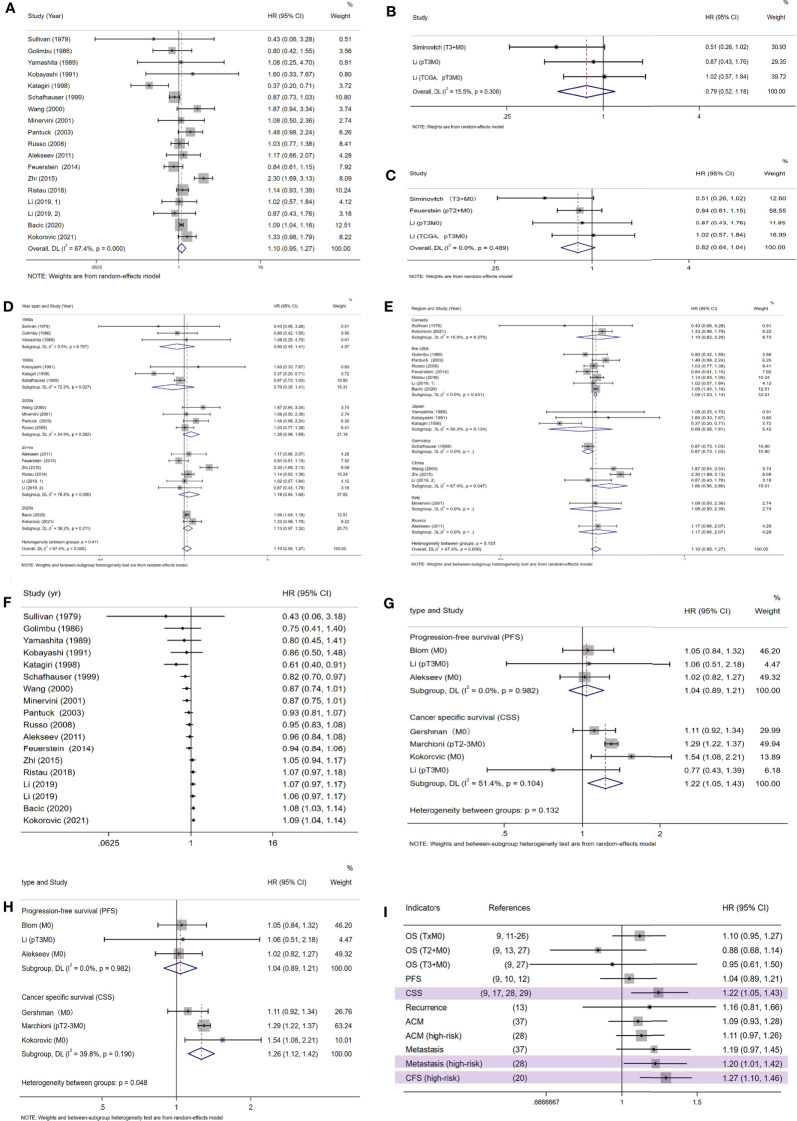
The meta-analysis results of survival outcomes with OS\PFS\CSS as indicators; **(A)** the meta-analysis result of RCC patients’ OS; **(B)** the meta-analysis result of OS of T3+M0 RCC patients; **(C)** the meta-analysis result of OS of T2+M0 RCC patients; **(D)** subgroup analysis according to the 10-year year span; **(E)** subgroup analysis according to the country where the research was carried out; **(F)** cumulative Meta analysis on OS; **(G)** the meta-analysis results of RCC patients’ PFS and CSS; **(H)** the results of PFS and CSS, with the literature of Li et al. excluded; **(I)** a summary table for evaluating the impact of LND on the prognosis of RCC patients with different survival indicators; OS, overall survival; PFS, progression-free survival; CSS, cancer specific survival; ACM, all-cause mortality; CFS, cancer-free survival.

**Figure 2A** showed the influence of LND on patients’ OS with non-metastatic RCC, indicating that LND did not influence patients’ OS (HR=1.10, 95%CI: 0.95-1.27). We further analyzed the influence of LND on T3+M0 (HR=0.79, 95%CI: 0.52-1.18) (**Figure 2B**) and T2+M0 (HR=0.82 95%CI: 0.64-1.04) (**Figure 2C**) RCC patients, which also showed no influence on the OS. Due to the heterogeneity indicated between studies with the Q test (p <0.001), we conducted subgroup analyses according to a 10-year span (**Figure 2D**) and country (**Figure 2E**).

**Figure 3:**
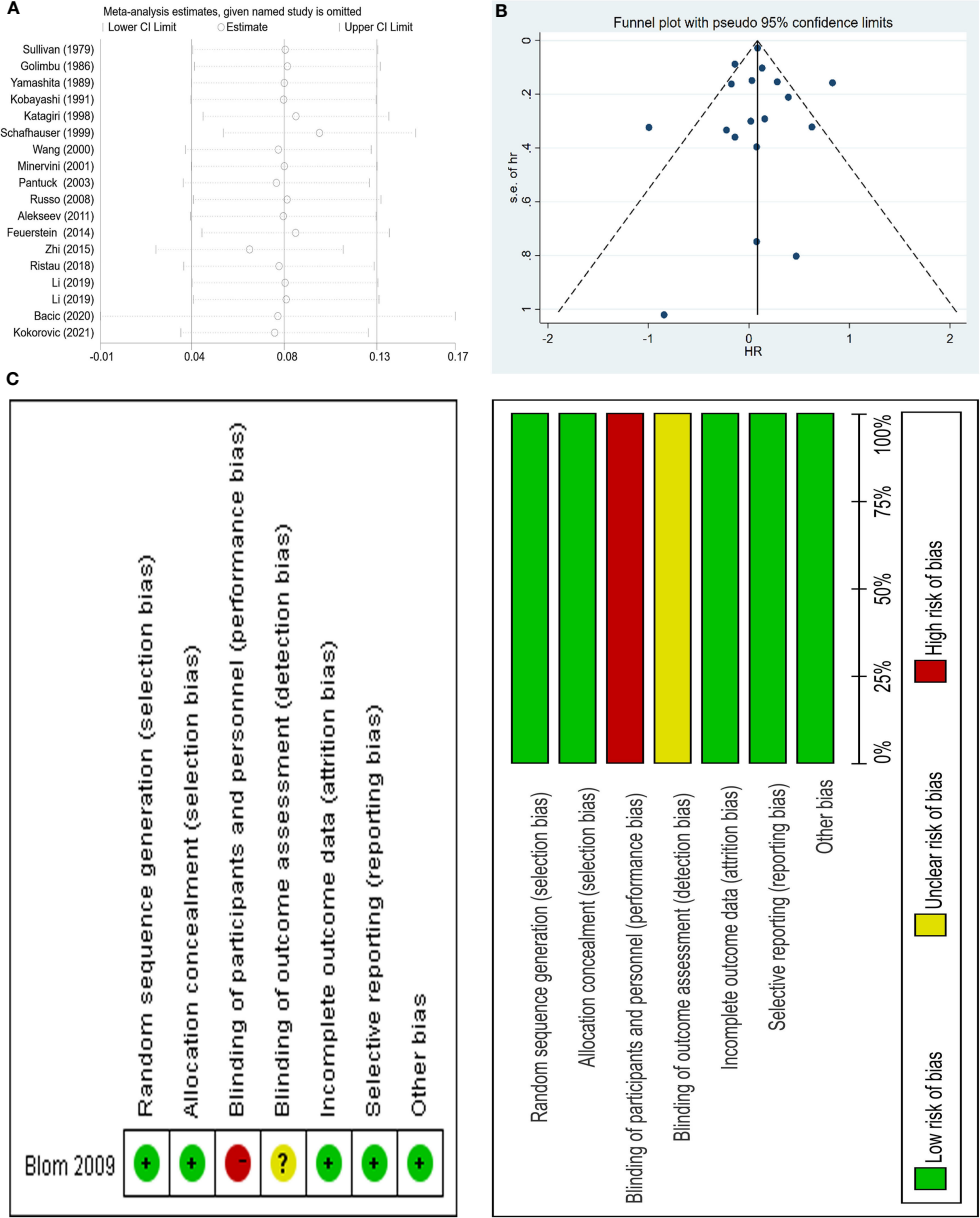
Sensitivity analysis and funnel plot of the meta-analysis OS as well as risk of bias summary of included trial; **(A)** sensitivity analysis; **(B)** funnel plot; **(C)** risk of bias summary of included trial.

And sensitivity analysis was done (**Figure 3A**). We tried to remove each study and did not observe significant difference, suggesting that our result was robust. No publication bias was found using the funnel plot (**Figure 3B**) as well as Egger test (p=0.974).

Finally, we conducted a cumulative meta-analysis, and interestingly, the results clearly and powerfully showed that the beneficial effects of LND on survival gradually diminished with the development of time (**Figure 2F**).

### Assessing Survival Benefit Through Other Survival Indicators

The effect of LND on PFS and CSS in patients with non-metastatic RCC was detected, with HR=1.04, 95%CI: 0.89-1.21 and HR=1.22, 95%CI: 1.05-1.43, respectively (**Figure 2G**). Due to the high heterogeneity within the CSS group, we excluded the literature of Li et al. and re-combined the effect size (HR=1.25, 95%CI: 1.14-1.37) (**Figure 2H**). The results showed that LND had no effect on patients’ PFS, but had a negative effect on CSS.

## Discussion

The incidence of RCC is increasing year by year with the advancement of imaging technology and the change of western lifestyle, with an incidence rate increasing by 2.421% annually (33, 34). According to GLOBOCAN data in 2018, it is estimated that 403,000 people are diagnosed with neoplasms of the kidney each year, accounting for 2.2% of all cancer diagnoses, with the highest incidence in North America (35). Among them, 175,000 people died of kidney cancer in 2018, accounting for 1.8% of global cancer deaths (35). It is expected that the disease burden of RCC in developing countries will increase in the coming decades.

For other types of urological cancers, LND is recommended, such as prostate and bladder cancer (36–39). Firstly, it can provide diagnostic value and help the selection of treatment options and prognosis judgment, that is, indirect benefit. Secondly, by removing the metastatic lymph nodes, it is possible to achieve a radical cure, that is, direct benefit. But when it comes to RCC, the value of LND on survival is still unclear. In 1969, Robson et al. advocated dissection of the para-aortic and paracaval lymph nodes from the bifurcation of the aorta to the crus of the diaphragm and believed that this retroperitoneal LND has improved the survival of patients (40). However, afterwards, various studies have not unified the survival benefits of LND. From 2010 to 2014, the trend of the proportion of LND for non-metastatic RCC patients showed did not change significantly (p=0.29), even in the subgroup of patients with cN+ disease, patients who received LND did not observe significant survival benefit (10). The only RCT study (Blom et al.) has great drawbacks, which only include low-grade (pT1-3) patients, and there is no evidence of cN+ before surgery (29). Therefore, the existing single research evidence cannot completely deny the survival benefit of LND for high-grade and cN+ subgroups. The latest 2019 guidelines only recommend the removal of clinically positive nodes, and the level of evidence is low (4). Although meta-analysis with similar content has been published in recent years, they have not conducted a separate analysis of the survival benefits of LND in patients with high-risk RCC, and the outcome indicators are limited to survival rate, and, more importantly, the large number of research results that have appeared in the past two years have not been included (41, 42).

**Figure 2I** shows a summary of results of different survival indicators. Based on the results of other single studies, we found that LND has no effect on the recurrence (27), metastasis (31), and all-cause mortality (ACM) of RCC patients (30, 31). But it presents as a risk factor for the metastasis of high-risk patients (30) and their cancer-free survival (CFS) (20). This is consistent with the results of this study. Our study has observed that LND has no effect on the OS of either TxM0 or T2+M0 or T3+M0 patients. However, when considering CSS, LND operation during nephrectomy is harmful. Given that CSS is a more meaningful clinical endpoint than OS and PFS, we believe that surgeons should carefully choose the target population of LND. Researchers have already found that patients receiving LND have larger, higher-stage tumors, which indicates a bias in surgical selection. The previously considered benefits of LND may only be due to this unidentified selectivity bias, rather than therapeutic effects. At the same time, taking into account the changes in the diagnostic criteria for RCC (for example, the infiltration of the pelvic system is placed under T3a in the updated AJCC TNM system standards), this reclassification may lead to a significant improvement in clinical results, the so-called statistical illusion of Will Rogers phenomenon. At the same time, LND may increase the operation time and blood loss of patients, and increase the risk of perioperative death and complications, for example, lower extremity edema, deep vein thrombosis, renal failure, adrenal insufficiency, chyloascites and intestinal obstruction ischemic colitis (29). Considering the above factors, we have reason to believe that the benefits of LND are even lower than the results of our research.

However, LND is not without benefit: On the one hand, in cT3-T4N0 or cN1 or cM1 patients, LND helps to obtain more accurate staging. On the other hand, for metastatic (M1) patients who are suitable for tumor reduction and potential follow-up systemic treatment, local LND surgery may be considered (4). The rationale for the potential oncological benefits of LND in RCC is based on the following premise: where the disease is limited to lymph nodes, complete resection may be curative and cytoreduction surgery may improve the response to systemic therapy and overall oncology results (43). Yu et al. found that for American Joint Committee on Cancer (AJCC) stage III patients, the OS and CSS of N1 were significantly lower than those of N0 patients, and even similar to those of stage IV patients (44). A large-scale retrospective study also found that the survival rates of patients with stage III and stage IV RCC with positive lymph nodes were similar (45). Therefore, the current trend is to reclassify RCC patients with nodal disease as stage IV disease (44, 45).

Thus, assessing the potential population that may benefit from LND act as an important and difficult priority for clinicians. Since the results of multiple studies agree that regardless of other prognostic factors, positive lymph nodes (N+) have clearly shown an independent adverse effect on tumor outcome, thus emphasizing the importance of how to predict lymph node metastasis clinically (46–48). It is worth noting that we did not conduct a subgroup analysis of high-risk patients, because different studies have very different limitations on high-risk patients. So how to define high-risk patients and selectively perform LND? Through a retrospective study of the NCDB database, Radadia et al. found that from 2010 to 2014, for patients with non-metastatic RCC, patients with cLN positive are more likely to undergo LND (49). Although Studer et al. used preoperative CT imaging to determine the status of cLN may not be related to the status of pLN (50). In addition, treatment center type, distance to treatment center, cT stage are also related to the increased risk of receiving LND (49). In practice, before surgery, clinicians can predict positive lymph nodes through imaging techniques (ultrasound, computed tomography [CT] and magnetic resonance imaging [MRI]). But it is not completely reliable. The minimum size of lymph node metastasis (less than 5mm) may be lower than the resolution of the machine, resulting in false negatives, and other lymphadenopathy may cause false positives (51). The Reporting and Data Systems (RADS) is no stranger to urologists, and quickly reminds people of PI-RADS for MRI detection of prostate cancer. Elsholtz et al. developed a Node-RADS-based standardized assessment method for cancer lymph nodes, the use of which can quickly guide imaging doctors to perform lymph node involvement in various cancer types including renal cell carcinoma based on CT or MRI images, which facilitates pre-operative standardized lymph node reporting (52). Moreover, there are currently various preoperative nomograms to predict lymph node infiltration (LNI), which is said to have an accuracy of 78.4% to 82.4% (53, 54). Similarly, we cannot ignore the prediction of lymph node metastasis using ancer Genome Atlas. miRNA-21 and miRNA-223 were found to be independently related to lymph node metastasis, with area under the curve (AUC)=0.738 (55). Interferon-induced transmembrane protein 2 (IFITM2), an inflammation related gene was reported to be associated with lymphatic metastasis and poor clinical outcome (56). During surgery, clinicians can rely on frozen section analysis of enlarged lymph nodes, with positive predictive value and negative predictive value of 100% and 95%, respectively (57). In general, we only recommend removing lymph nodes that are enlarged on imaging or that can be palpable during surgery.

The study by Kates et al. showed that among 37,279 RCC patients from 1988 to 2005, the amount of LND operations began to decline after 1988, and compared with the period of 1988-1997, the probability of LND procedure decreased significantly during the period of 1998-2005 (OR=0.65, 95%CI: 0.59-0.71) (58). Our cumulative meta-analysis result might could explain this phenomenon to a certain extent, which is because we have observed that since 1979, the benefit of LND for patient survival has gradually diminished over time, and even has a trend of reversal. This tendence of decline in effect of LND may be partly due to the rise of adjuvant therapy. The results of the first interim analysis of KEYNOTE-564 (NCT03142334) showed the benefits of pembrolizumab for disease-free survival (DFS)din high-risk patients (59). The Phase III PROTECT trial (insisted that dose intensity may matter as a DFS benefit among the 403 patients receiving 800 mg of pazopanib, yet no benefit in OS has yet been seen among the total cohort (60). PROSPER RCC (NCT03055013) is an ongoing Phase III randomized trial to evaluate the impact of nivolumab before and after nephrectomy through PD-1 blockade (61). Previous RCT has proven that in patients with advanced RCC who have previously received one or two regimens of antiangiogenic therapy, compared with everolimus, nivolumab has a longer OS and a lower incidence of adverse events (62).

As different studies have different definitions of patients with high-risk kidney cancer, we urgently need a method or model to accurately predict lymph node metastasis before surgery to explore the patient population that can really benefit from LND. At the same time, the scope and number of LND vary with surgeons in different countries. The standardization of LND templates is also one of the issues discussed in the future.

As far as we know, our study is the most comprehensive meta-analysis to date on the impact of nephrectomy combined with LND on the survival of patients. However, our research does have the following limitations. First, the limited RCT articles and prospective studies have made bias and heterogeneity widespread. It is difficult to balance the baseline characteristics among different original articles, including tumor stage, pathological type, and LND template, etc. And there is only one RCT article in this field. Second, we did not consider the impact of other variables on patient survival, that is, the surgical selection bias mentioned above and whether it is combined with postoperative adjuvant treatment as well as other confounding factors. Our article is an exploratory meta-analysis of this field. Through this article, we found that even though it is not beneficial for the survival of a wide range of RCC, the benefits of LND for the survival of medium-to-high-risk, high-risk and cN+ patients still need to be supplemented by more valuable evidence. Our meta-analysis provide a restrictive direction for subsequent original research. Thus, we call for more original researches on the role of LND in RCC, especially the emergence of RCT.

## Conclusions

LND has no survival benefit for all RCC patient groups and even high stage groups, and is even harmful to the patient’s CSS. However, for patients with positive lymph nodes before or during surgery, or assessment of high-risk patients, LND is of potential benefit. Large volumed and well-designed RCT articles are still needed to verify the patient population suitable for LND operations and good evaluation methods.

## Author Contributions

Conception and design, XS and DF. Administrative support, WW. Provision of study materials or patients, XS and DF. Collection and assembly of data, XS and DF. Data analysis and interpretation, XS and DF. Manuscript writing, all authors. Final approval of manuscript, all authors. All authors contributed to the article and approved the submitted version.

## Funding

This work was supported by Department of Science and Technology of Sichuan Province (2020YFH0099) The funders had no role in study design, data collection and analysis, decision to publish, or preparation of the manuscript.

## Conflict of Interest

The authors declare that the research was conducted in the absence of any commercial or financial relationships that could be construed as a potential conflict of interest.

## Publisher’s Note

All claims expressed in this article are solely those of the authors and do not necessarily represent those of their affiliated organizations, or those of the publisher, the editors and the reviewers. Any product that may be evaluated in this article, or claim that may be made by its manufacturer, is not guaranteed or endorsed by the publisher.
